# Predicted community consequences of spatially explicit global change‐induced processes on plant–insect networks

**DOI:** 10.1002/ece3.70272

**Published:** 2024-09-16

**Authors:** Hsi‐Cheng Ho, Florian Altermatt

**Affiliations:** ^1^ Department of Aquatic Ecology Swiss Federal Institute of Aquatic Science and Technology (Eawag) Dübendorf Switzerland; ^2^ Institute of Ecology and Evolutionary Biology National Taiwan University Taipei Taiwan; ^3^ Department of Evolutionary Biology and Environmental Studies University of Zurich Zurich Switzerland

**Keywords:** distribution fragmentation, functional homogenisation, global change, Lepidoptera–plant network, secondary extinction, trophic cascade

## Abstract

Plant–insect trophic systems should be particularly sensitive to processes altering species spatial co‐occurrences, as impacts on one level can cascade effectively through the strong trophic reliance to the other level. Here, we predicted the biogeography of Lepidoptera–plant communities under global‐change scenarios, exploiting spatially resolved data on 423 Lepidoptera species and their 848 food plants across the German state of Baden‐Württemberg (ca. 36,000 km^2^). We performed simulations of plant extinction and Lepidoptera expansion, and respectively assessed their cascading consequences—namely secondary extinction of Lepidoptera and change in functional distance of plants—on the interaction networks. Importantly, the simulations were spatially explicit, as we accounted for realistic landscape contexts of both processes: Plant extinctions were simulated as “regional” (a species goes extinct in the whole region at once) vs. “isolation‐driven” (a species gradually goes extinct from the peripheral or isolated localities according to its real regional distribution); Lepidoptera expansions were simulated with random, northward, and upward directions according to real topography. The consequences were assessed based on empirical community composition and trophic relationships. When evaluated by regional richness, the robustness of Lepidoptera assemblages against secondary extinctions was higher under isolation‐driven plant extinctions than regional plant extinction; however, this relationship was reversed when evaluated by averaged local richness. Also, with isolation‐driven plant extinctions, Lepidoptera at the central sub‐region of Baden‐Württemberg appeared to be especially vulnerable. With Lepidoptera expansions, plants' functional distances in local communities dropped, indicating a possible increase of competition among plants, yet to a lesser extent particularly with upward movements. Together, our results suggested that the communities' composition context at the landscape scale (i.e., how communities, with respective species composition, are arranged within the landscape) matters when assessing global‐change influences on interaction systems; spatially explicit consideration of such context can reveal localised consequences that are not necessarily captured via a spatially implicit, regional perspective.

## Graphical abstract

Landscape species‐composition context matters when assessing global‐change related changes on interaction systems, and spatially explicit simulations bring about localised consequences not necessarily captured via a regional perspective.

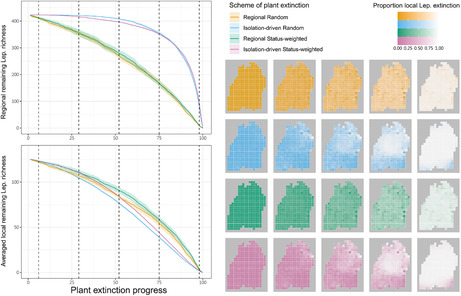

## 1 INTRODUCTION

Plants and insects together make up the majority of multicellular species diversity (Schoonhoven et al., 2005). Moreover, they are linked through a wide array of interactions, and such plant–insect interactions play key roles in numerous ecological processes and functions (Herrera & Pellmyr, 2009; Schoonhoven et al., 2005; Weisser & Siemann, 2008). For instance, insect herbivory regulates nutrient cycling, plant production and diversity in ecological communities; meanwhile, insect pollination is critical for the reproduction of a significant part of angiosperms. The understanding of plant–insect interactions has therefore long been a central ecological research pursuit, and is crucial for resolving and maintaining the integrity of biodiversity (Fisher, 1998; Samways, 1993).

Recent evidence suggests that global change is resetting the spatial and ecological equilibrium of interacting plants and insects (DeLucia et al., 2012; Pincebourde et al., 2017). Generally, global change drives two major aspects of species distribution: species change their distributional range and colonise new places, and species go locally extinct (Brook et al., 2008; Freeman et al., 2018; Thomas et al., 2006). The former is associated to species’ spatial movement to track their suitable or tolerable niches, while the latter by species’ inability to adapt to environmental changes. These influences thus present spatial processes that alter the distribution of species, as well as the composition of local communities. In the context of plant–insect interaction, effects on one (or multiple) species at one trophic level will cascade to the interacting others, creating trophic cascades between plants and their insect consumers (Dyer & Letoumeau, 1999; Jamieson et al., 2012). Such cascading effects should be particularly pronounced when the dependence on the respective other trophic level is strong, as is often the case in specific plant–insect interaction networks.

Lepidoptera (butterflies and moths) are one of the most widespread and species‐rich insect orders that exhibits such tight relationships (Scoble, 1995). Most Lepidoptera are exclusive plant feeders and rely two‐fold on plants across their life stages: their larvae are key herbivorous consumers to their host plants, whereas feeding adults pollinate their nectar plants (Boggs, 1987). Therefore, their trophic reliance on plants as a biotic filter shaping their spatial distribution is especially effective, on top of their climate niche as an abiotic filter (Nakadai et al., 2018). Consequently, Lepidoptera–plant interaction networks should be particularly sensitive to the alterations of species co‐occurrence, and present a suitable object for projecting possible cascading influences triggered by common global change‐induced spatial processes (Tscharntke & Brandl, 2004).

Two scenarios are of particular research interest: (i) resource plants go (locally) extinct, and (ii) Lepidoptera expand their range. While other scenarios (e.g., plant range expansion) are also valid, these two are well‐grounded on evidence and expected to happen relatively quickly (Hällfors et al., 2021; Suckling et al., 2017). Loss of local plant occurrences is an important part of the ongoing biodiversity crisis, as about 40% of the world's plants are at risk of either local or global extinction (Nic Lughadha et al., 2020). The robustness of Lepidoptera–plant communities—as interaction networks—responding to this change (or not) through trophic cascade is thus a critical feature to be understood (Banza et al., 2015; Pearse & Altermatt, 2013a). Being able to predict the response of such Lepidoptera–plant networks helps to identify the Lepidoptera that are prone to secondary extinction due to losing food. Meanwhile, adult Lepidoptera are generally highly mobile and able to change their range rapidly (Neve et al., 1996). Tracking climate niche under climate change is considered a main trigger of the reported Lepidoptera expansion (Hill et al., 2021). Notably, theory has specifically suggested that the “warm” boundary of spatial distribution is often driven more by a species’ intrinsic climate niche (e.g., temperature tolerance), while the “cold” boundary by its biotic interactions (Paquette & Hargreaves, 2021). In a warming world, Lepidoptera are expected to expand their cold boundaries and reach places where they have not been, forming interactions with the locals, thereby creating a need to account for the bio‐interaction context to better predict the consequences of such expansion. For instance, in the absence of diet shifts, successful Lepidoptera range expansion depends on the availability of respective food plants in the new range, and may alter the trophic structure and functions of the communities therein.

To resolve, and further to predict possible influences of global change on Lepidoptera–plant communities, here we exploit extensive empirical data of a real‐world landscape to explore in silico the consequences of the two focal spatial processes, namely the secondary extinction of Lepidoptera caused by plant extinction, and the change in functional relationships among plants caused by Lepidoptera expansion. Importantly, while relevant previous studies are mostly spatially implicit (e.g., Pearse & Altermatt, 2013a), our simulations are spatially explicit and based on simple but realistic information of the topography, species occurrences, and interactions within the landscape (echoing Tscharntke & Brandl, 2004), thereby integrating empirical biogeographical and bio‐interaction knowledge. As for plant extinction, we specifically compare between regional (spatially implicit null) and distributional isolation‐driven (spatially explicit) schemes. In analogy, for Lepidoptera expansion, we compare among random shifting (spatially implicit null) and directional (northward and upward, spatially explicit) schemes, respectively. Their community‐wise consequences, including secondary extinction and functional changes, are evaluated with realistic community composition and trophic structure resolved to the local scale. We expect that spatially explicit simulations will bring about more constrained yet arguably more realistic consequences than the nulls, and those consequences tend to be negative to local communities. Also, we expect that spatially explicit schemes will reveal certain localised patterns of response shaped by the realistic correspondence of communities.

## 2 MATERIALS AND METHODS

### 2.1 Study area

Our study area is the German state of Baden‐Württemberg, which spans roughly 36,000 km^2^ and elevation‐wise 85–1493 m above sea level. The elevational and biological data we used (see further below) were spatially resolved to a 10′ longitude × 6′ latitude (roughly 10 × 10 km^2^) grid and covered 302 such cells. Correspondingly, we hereinafter refer to such cells as the “local” scale at which individual ecological community resides, while the whole area (all cells altogether) as the “regional” scale that represents the landscape. Data of the mean elevation of each cell were extracted from the GIS database *Shuttle Radar Topography Mission* (*SRTM*, NASA/NGA).

### 2.2 Lepidoptera–plant communities and interactions

This study considered Lepidoptera and plant species whose occurrences were recorded between 1985 and 2014 in the study area. All Lepidoptera are native to that region, and the plants considered cover all plants to be found in the wild, including native, non‐native, and naturalised ornamental plants and crops. These occurrence data were derived from respective long‐term empirical surveys covering all the focal cells (Database *Arbeitsgruppe Schmetterlinge Baden‐Württembergs am SMNK* (https://www.schmetterlinge‐bw.de) for Lepidoptera; *Bundesamt für Naturschutz 2010* and *Floraweb* for plants), and we further compiled them into simple presence‐absence status of species per cell across the selected three‐decade time window. The surveys were not strictly systematic but somewhat haphazard, but the accumulative effort was very intensive (with totally over 74,000 yearly cell‐wise occurrence entries for Lepidoptera and 510,000 for plants, each of which was already an aggregation of many observation records), so the compiled cell‐wise occurrences should representatively inform the composition of each local Lepidoptera–plant community at the spatial and temporal scales we looked at. For Lepidoptera, we regarded that a species’ occurrence represents both of its life stages within a given cell. We note that, collapsing three‐decade survey data into one presence‐absence occurrence status improves the completeness of our understanding on communities' composition, meanwhile presents an empirically‐bounded initial state for our subsequent simulations (see further below); yet, this operation overlooks that species distributions may had been changing within the time window, and it may overestimate Lepidoptera's spatial distribution by counting in some transient occurrences. Nonetheless, we decided to temporally collapse the occurrences this way because (i) we did not possess additional information allowing us to judge the transiency of occurrence entries; (ii) the data showed that alternative collapsing intervals made little difference in terms of detecting species richness in the study area (Figure S1); (iii) future distributional changes (i.e., what we would like to simulate) are supposed to be more pronounced than past ones (i.e., what might be lost in our compiled occurrences) given global‐change influences are accelerating (Elsen et al., 2022); therefore, a slightly overestimated initial state shall not override the consequences we may detect with simulations, as long as the simulated distributional changes of species are set to be substantial (see further below).

We subsequently compiled the Lepidoptera's dietary information, including their larval usage of host plants and adult usage of floral resources, based on the work of Ebert (1991–2005) and further complemented with additional references (Altermatt & Pearse, 2011; Pearse & Altermatt, 2013a, 2013b, 2015). All these dietary interactions between Lepidoptera and plants were based on empirical observations under natural situations in the field within the study area, and were recorded by professional entomologists over more than 50 years, accumulatively with more than 2.3 million records. From this, we extracted the binary dietary relationship (i.e., interacting or not) between the Lepidoptera and plant species, larval and adult stages separated. We did not infer interaction strengths or preferences because the multiple data sources did not inform such information to equal extents nor in similar format. The binary‐coded interaction is therefore a general, conservative foundation for judging Lepidopterans’ edible diets, and is coherent to the logic of subsequent simulations where Lepidoptera secondary extinctions are driven by lacking food (see further below).

With the compiled dietary information, we applied a “metaweb” approach (also “metanetwork”, referring to a network formed by all known interactions among the focal species; see Ho et al., 2022; Saravia et al., 2022; Thuiller et al., 2024) to inferentially construct local Lepidoptera–plant interaction networks from their cell‐wise co‐occurrences. The approach assumes that each Lepidoptera species at a given life stage, at least within the spatial scope of our study area, has a fixed diet that is already captured by our compiled dietary information. We further assumed no spatial structural nor mobility restrictions of species within a cell, so co‐occurring species can locally encounter and interact with each other. In other words, a Lepidoptera–plant interaction that was ever recorded at a certain cell will realise also elsewhere in the focal landscape as long as the corresponding insect and plant are locally co‐occurring. Such assumptions implicitly acknowledge that these dietary interactions are driven by functional trait matching (i.e., the “edibility” of the food; driven by, e.g., chemical tolerance in Lepidoptera, see Després et al., 2007) and bounded by empirically recorded interactions, and collapse all potential intraspecific variations at the species level (Ho et al., 2022). We note that plasticity, acclimation, and potential adaptation in diets were thus excluded from the scope of our simulations, though these mechanisms may have contributed to the formation of local interaction networks in the real world. Also, we might overestimate realised interactions at our spatial scale of cells if certain Lepidoptera and their recorded co‐occurring food plants were actually locating non‐overlappingly within a cell. Such overestimation should be of minor extents, though, given the high mobility of Lepidoptera adults.

Based on our data‐grounded occurrences and dietary metaweb, we examined a subset of the real Lepidoptera–plant communities. That is, species with locally realised interactions (i.e., species without local interacting partners were excluded) and, for Lepidoptera, those having food plants locally available for both life stages (i.e., species without locally co‐occurring food plant at either life stage were excluded). We applied these criteria throughout the region, thereby our study coherently looked at the same subset of species, and the networks they formed across space. From the overall 980 Lepidopterans and 3147 plants recorded occurring, our following simulations focused on 423 Lepidopterans and their 848 food plants that were involved in local interaction networks spatially spanning the 302 cells. With these focal species, the potential diet breadths (in terms of number of edible food plants listed in the metaweb) of larval Lepidopterans ranged 1–69 with median 3, and that of adult Lepidopterans 1–203 with median 3 (histograms see Figure S2).

### 2.3 Global change‐induced spatial processes simulation

Starting with all the local Lepidoptera–plant networks as above addressed, we simulated the focal spatial processes—plant extinction and Lepidoptera expansion—in the study region. All simulations therefore shared the same initial state, and had consequences emerged from the same regional trophic structure, both given by empirical data.

**FIGURE 1:**
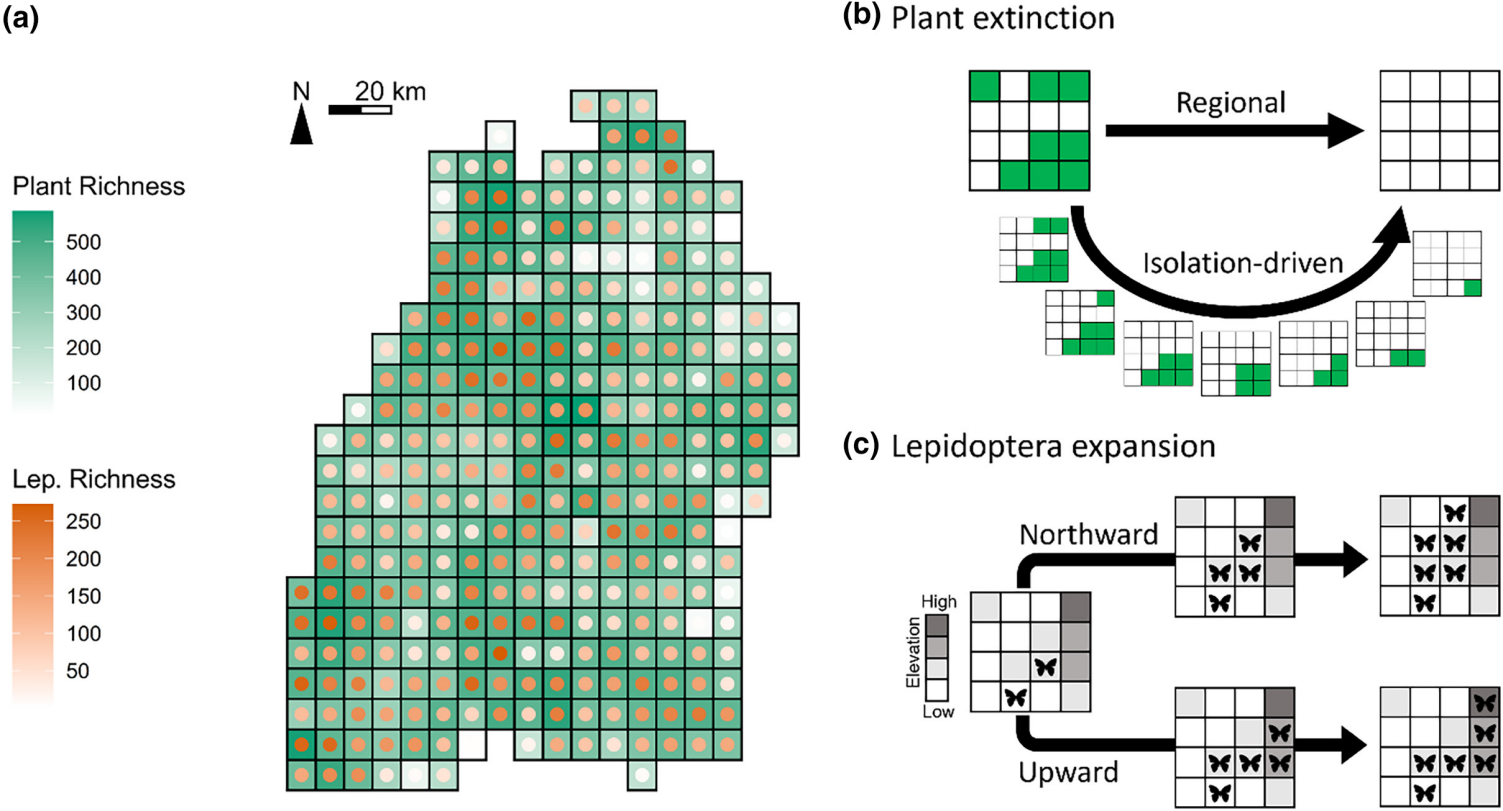
(a) Richness of plants and Lepidoptera in the study area at the initial state (before simulation) given by empirical data. (b) An exemplary illustration of the two ways of simulating plant extinction, spatially implicit “regional” and spatially explicit “isolation‐driven”. (c) An exemplary illustration of the two ways of simulating directional Lepidoptera expansion, orientational “northwards” and elevational “upwards”.

**FIGURE 2:**
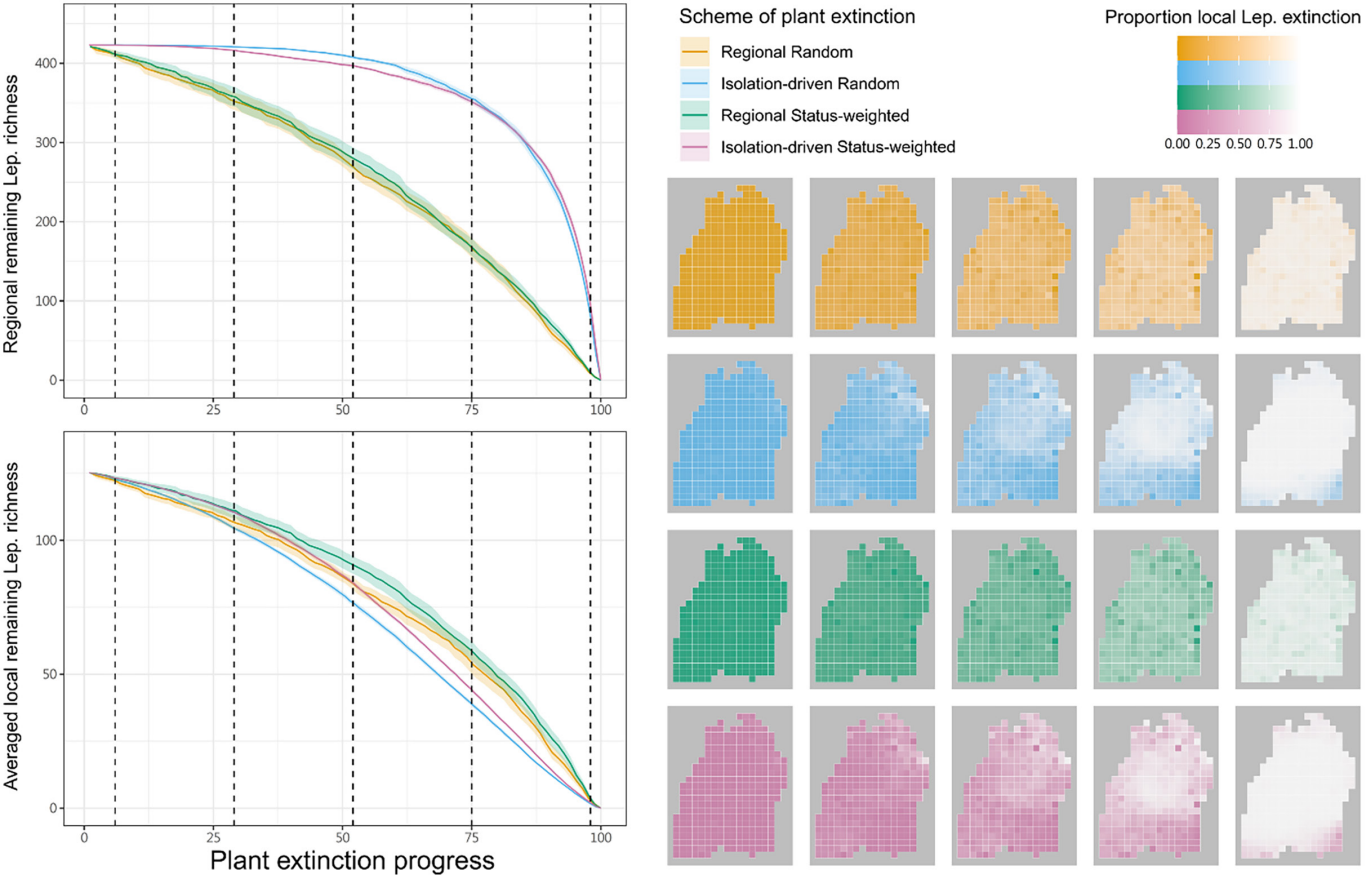
Lepidoptera's secondary extinction due to simulated plant extinction across the four schemes (indicated with colours). The left panel presents regional richness and averaged local richness of remaining Lepidoptera throughout the course of simulations, respectively. The shaded areas present the 95% CI across 20 replicates. The proportion (relative to the initial state) of local Lepidoptera that has gone extinct at five selected time points (vertical dashed lines in the left panel) was visualised as five corresponding columns of maps at the right panel, all averaged across 20 replicates.

For plant extinction, we first looked at two main schemes, namely “regional” and “isolation‐driven”. With the former, at each simulation step, a plant species (from the 848 focal food plants) was randomly selected and set extinct across the whole region, i.e., being removed from all the local communities where it originally occurred. This was therefore a spatially implicit, and arguably unrealistic, simulation of plant extinction. With the latter, again a plant species was randomly selected at each step, yet its extinction only happened locally at the most‐isolated or most‐periphery cell(s) according to the selected plant's current regional cell occupation (Figure 1; for detailed algorithm see Supplementary Information Section S1). A plant species thus must be selected several times to become fully extinct in the region. The isolation‐driven scheme thus incorporates and stresses, from a biogeographic perspective and in a spatially explicit manner, that isolated or periphery populations are often at higher extinction risk (Channell & Lomolino, 2000). Besides the above, to consider that certain species‐level features (in addition to spatial isolation) may differentiate the extinction likelihoods among plants and lead to different extinction dynamics, we also simulated another two schemes mirroring the previous two, but with the plants' status of being threatened integrated as a weighting factor to plant–extinction simulations (“status‐weighted”, in combination with “regional” or “isolation‐driven”). That is, the sequence of plants' going extinct became no longer purely random; highly‐threatened plants tended to go extinct early (details see Section S2). For all four schemes, after each step, we locally removed Lepidoptera species that lost completely either its larval or adult food plants due to the simulated plant extinction according to our metaweb, and such procedure presented the trophic cascade‐driven Lepidoptera's secondary extinction due to the lack of food. The simulations ended when all 848 focal plants (and thus all 423 interacting focal Lepidoptera) are fully extinct in the region, and we carried out 20 independent simulations for each scheme. Although different schemes required computationally different numbers of steps to reach the final state, the simulation steps per se were not temporally meaningful but only reflecting extinction events, thus the consequences (e.g., trajectories of secondary extinctions) of the schemes can be compared altogether given their shared initial and final states being aligned (Figure 2).

For Lepidoptera expansion, in analogy, we first looked at two main schemes that phenomenologically mimic the shifting of Lepidoptera cold boundaries: orientational “northward” and elevational “upward”. With the former, at each simulation step, we randomly picked 50 percent (a fixed parameter) of the Lepidoptera at each cell, and their occurrences were added to the cell at the adjacent north. With the latter, it was the same process yet the occurrences of the picked Lepidoptera were added to whichever of the eight adjacent cells having higher mean elevation than the original. Here we again additionally accounted for species‐level features that may influence the likelihood of Lepidoptera's expansion, thereby having another two schemes where Lepidoptera species‐specific flying ability (proxied by inversed wing‐load (Ebert, 1991–2005)) as a weighting factor (“wingload‐weighted”, in combination with “northward” or “upward”). That is, the expanding 50% local Lepidoptera were no longer randomly picked at each step; instead, better flyers were more likely to expand (Section S3). Notably, northward expansion was cell‐wise one‐to‐one per step, while upward expansion could be one‐to‐several. The per‐step spatial extents of Lepidoptera expansion were thus inequal between northward and upward (and corresponding wingload‐weighted) schemes. To set a baseline allowing across‐scheme comparisons, we also performed two spatially‐irrelevant null schemes. With these two nulls, the expanding Lepidoptera did not go to adjacent cells but randomly selected one(s), thus the spatial contexts of expansion were discarded. Meanwhile, the nulls respectively accounted for the number of Lepidoptera‐receiving cells at each step of the norward and upward simulations, thus kept the same spatial extents of expansion corresponding to respective northward or upward schemes. For all six schemes, an expanding Lepidopteran could only successfully establish in the new cell(s) if food plants were therein available supporting its both life stages, otherwise was removed instantly; in other words, lacking local food at the destination would impede its colonisation and range expansion. Throughout the simulations, some expanded Lepidoptera may already exist in the new cell(s) before expansion so would not lead to any compositional changes, while others changed the Lepidoptera composition in local networks. The (non‐null) schemes thus explicitly covered compositional (thus interactional), orientational, and topographical spatial contexts of Lepidoptera expansion. We simulated all schemes for three consecutive steps, for 20 independent replicates, and presented the consequences after the final step (other steps see Section S4).

In the above‐mentioned simulations, all extinction and expansion events that may change local Lepidoptera–plant communities’ composition operated at the spatial scale of our cells, which could be seen as a discrete approximation of the realistic gradual distribution changes of species over time. Consequently, the consequences of each simulation step should be relatively substantial, as they already reflected an integration of continuous processes over an implicit period of time.

### 2.4 Metrics and analyses

For plant extinction, we quantified the extent of Lepidoptera secondary extinction at each cell by counting the remaining number of Lepidoptera species after each simulation step. We derived the step‐by‐step proportion of local (per‐cell) Lepidoptera that have gone extinct, and summarised at the landscape scale the regional remaining (i.e., union of species across all cells; the gamma richness) and averaged local remaining (i.e., cell‐wise alpha richness averaged across all cells) Lepidoptera richness, which then were used to construct the Lepidoptera's secondary extinction trajectories. We also regressed the proportion of local Lepidoptera extinction to the corresponding cell's mean elevation, to check if local secondary extinction had any elevational association. Since local Lepidoptera secondary extinctions change the regional distribution of these species, we quantified the extent of distributional fragmentation of Lepidoptera as a follow‐through consequence of plant extinction. This was quantified as the distributional disconnectivity per Lepidoptera per step—that is, according to the focal Lepidoptera's current cell occupancy, for each occupied cell we derived the number of non‐connected other occupied cells, then summed the values and normalised to a maximum reference value derived as if every cell was disconnected from each other. Because looking individually at all 423 Lepidoptera species’ fragmentation change is impractical, we focused on three selected sets of 40 Lepidoptera species, composed by those having the broadest, the narrowest, and intermediate original spatial distribution in terms of cell occupation. By thus, we compared the fragmentation change trajectories among these sets, looking for potentially different fragmentation responses. These metrics were compared among plant extinction schemes across the whole simulation process.

As a consequence of Lepidoptera expansion, locally newly realised Lepidoptera–plant interactions were predicted after each step of simulation. We evaluated the normalised functional distance of plants and dietary niche overlap of Lepidoptera in these local interaction networks, to check how these community‐wise functional features may be changed caused by the joining of expanded Lepidoptera. The functional distance of plants was based on the binary coding of trophic interactions in local communities with regards to Lepidoptera identities, and calculated as the normalised sum across plants of Euclidean distance matrix evaluated by the *dist* function of inbuilt R package *stats* (v4.3.1). A low functional distance thus indicates that the plants in the local assemblage are interacting with relatively alike assemblage of Lepidoptera, and vice versa. The niche overlap of Lepidoptera was calculated using the *networklevel* function of R package *bipartite* (v2.18; Dormann et al., 2009), in terms of the composition of their local plant diets. A low niche overlap thus indicates that the Lepidoptera in the local assemblage have more differentiated diets, and vice versa. Notably, as Lepidoptera's larval and adult interactions with plants are intrinsically different, these metrics were evaluated and viewed separately with life‐stage‐specific sub‐networks. These metrics were compared among Lepidoptera expansion schemes (including the spatially implicit null models).

Note that, with simulative data, we refrained from reporting our results based on p values. In the following, all comparisons among schemes will be described qualitatively, yet were judged quantitatively with box plots or trajectories with respective confidence intervals based on observed variances under a fixed 20‐replicate condition (a conservative size to avoid creating artificial significance; see White et al., 2014). All simulations, quantifications and analyses were performed using R language (v4.3.1; R Core Team, 2013), and the script reproducing all tasks can be accessed at the provided repository.

## 3 RESULTS

**FIGURE 3:**
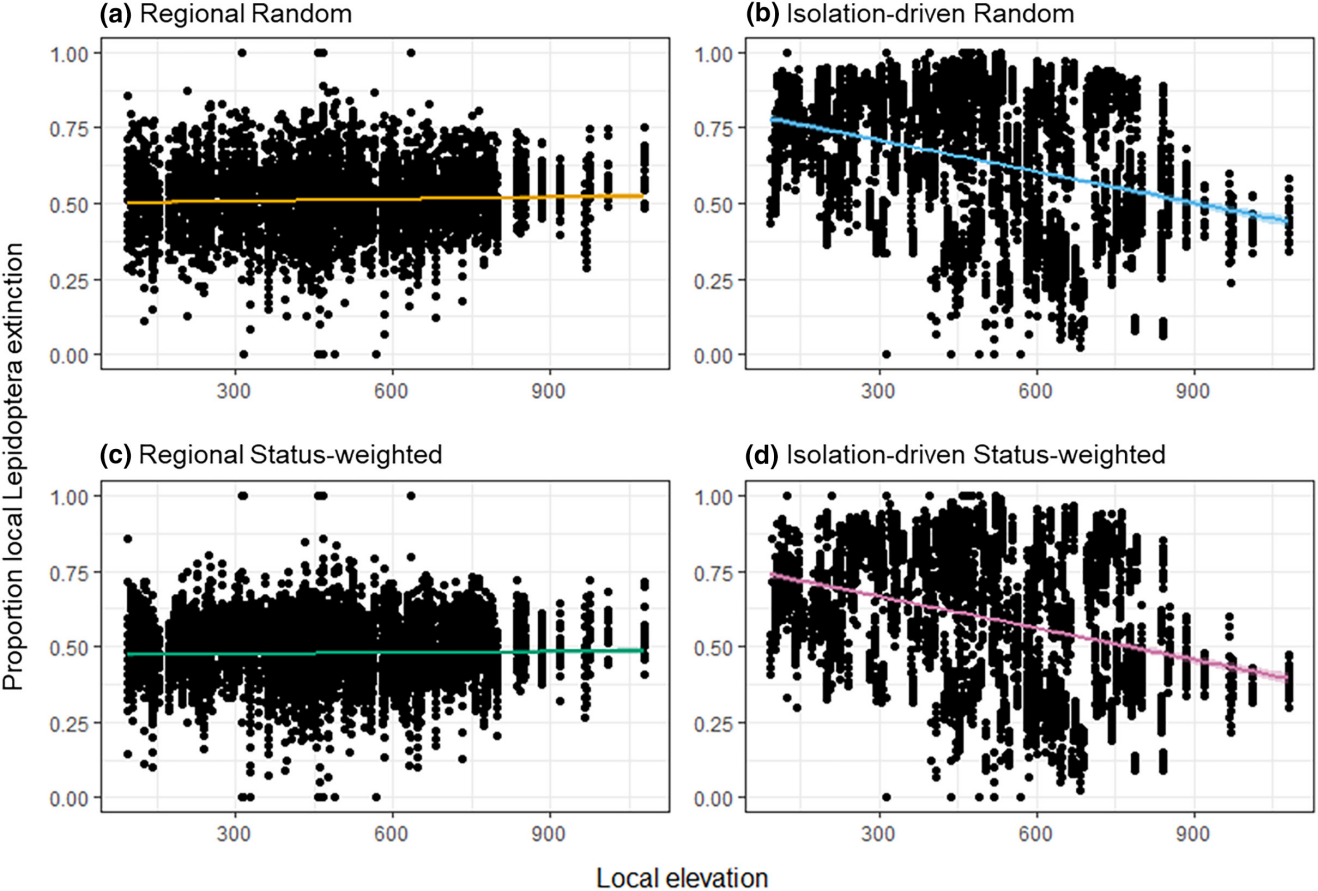
Proportion local Lepidoptera secondary extinction against the cell's local mean elevation in the four schemes of plant extinction simulation. These plots are at the states of the 4th selected time point (as in Figure 1); for other steps, see Figure S3. Here we aim at highlighting the higher Lepidoptera extinction rate associated with lower elevation specifically in isolation‐driven schemes, but not the regional ones; this was visualised using linear regression (coloured lines and shaded 95% CIs, across 20 replicates), while corresponding stats (including alternatives with mixed model setting replicates as a random effect) are provided in Table S1.

The predicted consequences of accumulating regional plant extinction on regional and averaged local Lepidoptera richness were similar, leading to gradual and smooth decreases in remaining richness, where the status‐weighted schemes kept higher Lepidoptera richness than the non‐weighted schemes (Figure 2). The proportion of local Lepidoptera extinction indicated that the rates of secondary extinction were rather even across grid cells (Figure 2). Contrarily, with isolation‐driven plant extinction (status‐weighted or not), regional Lepidoptera richness barely decreased in the first half of the simulations then exhibited an accelerating drop in the second, while averaged local Lepidoptera richness still exhibited gradual and smooth decreases (Figure 2). Throughout the simulations, the isolation‐driven schemes kept having more regional remaining Lepidoptera than the regional schemes; however, this pattern reversed when looking at averaged local remaining Lepidoptera. Between the isolation‐driven schemes, random extinction kept slightly more regional remaining Lepidoptera than status‐weighted extinction, yet the reverse when regarding local remaining Lepidoptera (Figure 2). With isolated‐driven plant extinction, the middle part of the landscape turned out to be particularly vulnerable to secondary extinction, i.e., with higher proportion of local Lepidoptera extinction than other areas, particularly later in the simulations (Figure 2), and such vulnerability was associated with lower elevation (Figure 3; Figure S2).

**FIGURE 4:**
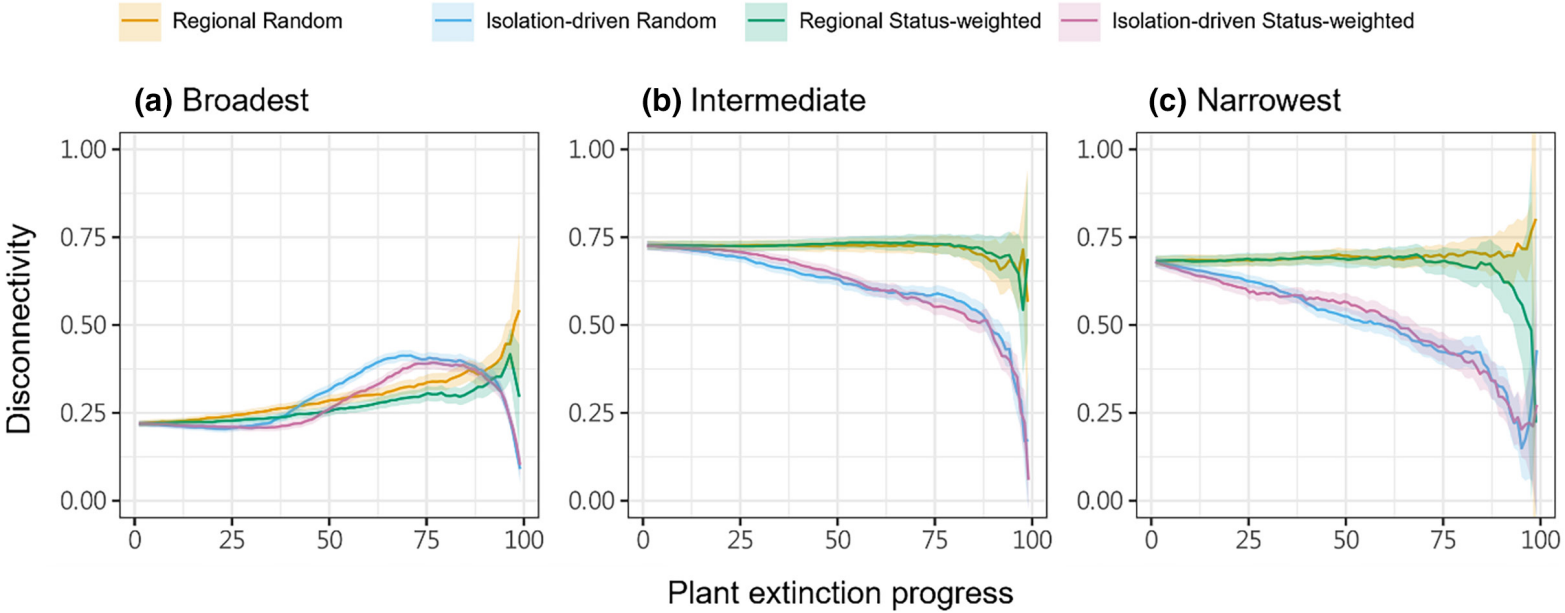
Lepidoptera distributional fragmentation quantified as disconnectivity along the four schemes of plant extinction simulation (indicated with colours). We present the trends derived respectively from the 40 Lepidoptera species that are originally broadest‐distributed (a), intermediately distributed (b), and narrowest‐distributed (c), judged by the number of cells occupied.

Lepidoptera's secondary extinction at individual cells further changed their landscape‐wise distributions. With regional plant extinction, the distribution of Lepidoptera that were originally broadly distributed (and relatively non‐fragmented) within our study landscape became gradually fragmented, while that of those originally intermediately or narrowly distributed (and already relatively fragmented) remain unchanged until very late phase of simulations (Figure 4). Meanwhile, with isolation‐driven plant extinction, broadly distributed Lepidoptera first had minimal response, then became more fragmented, further then more intact; instead, intermediately and narrowly distributed Lepidoptera gradually became more intact (Figure 4).

**FIGURE 5:**
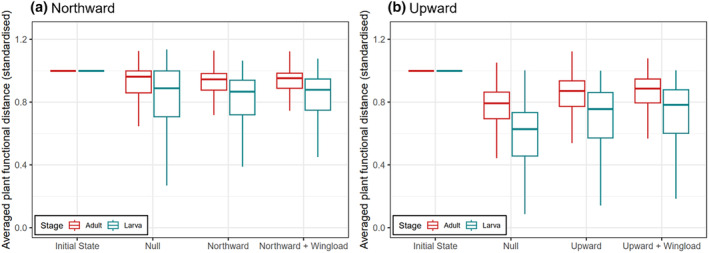
Averaged plant functional distance in the local interaction networks formed with adult and larval Lepidoptera (indicated with colours) at the initial state (before simulation) and after three steps of Lepidoptera expansion simulation. (a) panel shows northward, northward wingload‐weighted, and corresponding null schemes, and (b) panel shows the upward ones. The readings were standardised by dividing by the value at the initial state to highlight the relative changes; for original values, see Figure S4, for other steps, see Figure S5 & S6. The boxplots indicate the median (middle thick line), the 1st and 3rd quantiles (Q1 and Q3, the hinges of the box), and the smallest/largest value (the whiskers) within Q1‐1.5 × IQR and Q3 + 1.5 × IQR (where IQR = Q3‐Q1), respectively.

**FIGURE 6:**
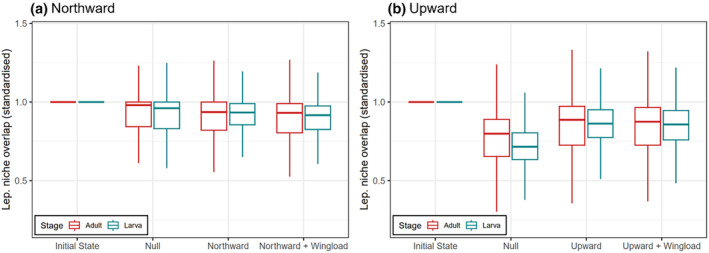
Averaged adult and larval (indicated with colours) Lepidoptera dietary niche overlap in the local interaction networks formed with food plants at the initial state (before simulation) and after three steps of Lepidoptera expansion simulation. (a) panel shows northward, northward wingload‐weighted, and corresponding null schemes, and (b) panel shows the upward ones. The readings were standardised by dividing by the value at the initial state to highlight the relative changes; for original values see Figure S7, for other steps see Figure S8 & S9. The boxplots indicate the median (middle thick line), the 1st and 3rd quantiles (Q1 and Q3, the hinges of the box), and the smallest/largest value (the whiskers) within Q1‐1.5 × IQR and Q3 + 1.5 × IQR (where IQR = Q3‐Q1), respectively.

As for Lepidoptera expansion's influences on the functional features of communities, in general, the nectar plants in local imago–plant networks were functionally more distant than the host plants in larva‐plant networks, which was consistent before (given by empirical data) and after simulated expansion (Figure 5; Figures S3–S5). The plants' functional distance dropped as a result of Lepidoptera expansion no matter it was northward or upward, but particularly in the upward schemes the dropping was weaker than the corresponding null (Figure 5). The functional distance of host plants in larval networks dropped slightly more than the nectar plants in adult networks (Figure 5). Spatially speaking, we observed no obvious patterns of any sub‐region having particularly intensive functional changes (Figures S4 & S5). The outcomes of wingload‐weighted and corresponding non‐weighted schemes were very similar, thus expansion at random or depending on (inferred) flying ability made little difference to the consequences of Lepidoptera expansion (Figure 5). The analogous results regarding Lepidoptera niche overlap were qualitatively mirroring those regarding functional distance (Figures 6, S6–S8).

Overall, our expectations were partially realised. Spatially explicit isolated‐driven plant extinction indeed led to more constrained regional Lepidoptera secondary extinction, and northward and upward Lepidoptera expansion brought about more constrained plants' functional distance changes, compared to respective, spatially implicit null models. A localised (sub‐regional) pattern of vulnerability to Lepidoptera secondary extinction was also revealed by isolated‐driven plant extinction. Yet, some metrics reflected stronger consequences in spatially explicit schemes than in implicit ones, for example, the averaged local Lepidoptera secondary extinction across the whole landscape.

## 4 DISCUSSION

We predicted possible consequences of key global change‐induced processes—plant extinction and Lepidoptera expansion—on the richness, composition, and trophic structures of lepidoptera–plant communities using spatially explicit simulations considering a real‐world topography.

When plants’ extinction scenarios were (distributional) isolation‐driven, Lepidoptera were more robust to secondary extinction if evaluated by regional Lepidoptera richness, but less robust if evaluated by averaged local Lepidoptera richness, in comparison to the case of regional plant extinction. This is reasonable, as with isolation‐driven extinction, a plant gradually disappears from individual cells where it occurs. Correspondingly, should this plant's extinction trigger any secondary extinction of its Lepidopteran consumers, it would be also gradual and local, and thus can only be detected via local but not regional richness of Lepidoptera. If isolation‐driven plant extinctions were status‐weighted, compared to non‐weighted ones, they led to less robust regional richness but more robust local richness of Lepidoptera. This is likely because the status of being threatened evaluates multifaced properties of plants, not only abundance but also distributional ones, such as the level of fragmentation (Section S2). Thus, compare to the random case, status‐weighted extinction tends to make threatened plants that are already fragmented/isolated experience local extinction earlier in the simulations, quickly resulting in these plants’ regional extinction; correspondingly, we saw a faster accumulation of Lepidoptera regional extinctions. Meanwhile, threatened plants are already scarce, and the Lepidoptera‐plant communities constructed with empirical observations should have (quasi‐)equilibrated at conditions where these threatened plants do not (or no longer) play critical trophic roles. Thus, the Lepidoptera impacted by their local extinctions should be comparatively fewer, and correspondingly we saw a slower accumulation of Lepidoptera local extinctions. Importantly, different spatial perspectives may provide different, sometimes even opposite, predictions of the communities’ biogeographic change. Even when addressing processes at the landscape scale, localised contexts (e.g., trophic structure) and responses (e.g., local species loss) should not be neglected, as biodiversity management may require fine‐tuned, multi‐scalar practises (Ballare et al., 2019).

Adding to our previous understanding of Lepidoptera in this area (Habel et al., 2019; Karbiener & Trusch, 2022; Ho et al., 2024), isolation‐driven plant extinction revealed that the central part of Baden‐Württemberg, at lower elevation, was more prone to Lepidoptera secondary extinction. We see two possible mechanisms: (i) the localised, original trophic structure of the communities in this sub‐region were more prone to secondary extinction, e.g., many Lepidoptera fed on a few plants; (ii) the isolation‐driven plant extinction targeted more frequently at the cells in this sub‐region. With the former, regional plant extinction schemes would have also generated the same spatial pattern of secondary extinction, yet this was not the case, making the latter more likely. This sub‐region of Baden‐Württemberg has low forest coverage and relative intensive agriculture (Petig et al., 2019; Schindler et al., 2009). As intensive agriculture has been recognised to cause habitat fragmentation of species by breaking the continuity of natural distribution (Walker et al., 2009), it is unsurprising that our focal food plants distributed as relatively isolated cells within this sub‐region (Figure S10). Notably, plants' distributional isolation was evaluated at the resolution of our grid cell (10 × 10 km^2^) constrained by our biological data.

Local secondary extinction of a Lepidopteran may make its regional distribution less fragmented by removing some already isolated cells, or more fragmented by disconnecting some larger patches of cells. Particularly for broadly distributed Lepidoptera under isolation‐driven plant extinction, we saw both happened, as their level of fragmentation first changed minimally, then increased relatively rapidly, and then dropped in the late phase of the simulation. Intermediately and narrowly distributed Lepidoptera may not have large enough original distribution to allow the formation of disconnected patches, thus could not respond as variably. Distribution fragmentation (and the likely resultant small populations) is known to associate with impeded genetic flows (Kwak et al., 1998; Williams et al., 2003) and vulnerability to extinctions driven by ecological drift (Gilbert & Levine, 2017). Therefore, beyond our estimated secondary extinctions caused by trophic cascade, plant extinction may impose additional harm to the Lepidoptera (especially broadly distributed ones) via disrupting their population interaction. We note that, however, some patterns we report here occurred only in the later phase of simulations, after half of the focal food plant has gone extinct. Such a devastating level of plant loss would come along with whole ecosystem's malfunction, and be unrealistic to be focused through our lens of effects on insect‐plant networks only.

In general, plants’ functional distance decreased as Lepidoptera expand. This implied that apparent competition among plants via sharing herbivores became stronger in larva‐plant networks, and so did direct competition over pollinators in imago‐plant networks. Thus, via altering the local trophic structure at the arrived communities, Lepidoptera expansion may modify the plants' local competitive structure (sensu Orrock et al., 2010; Rand, 2003). Though the observed extent of functional distance drop was larger in the upward than the northward expansion schemes, this was due to the larger per‐step extent of expansion of the former; comparing with respective nulls actually showed the opposite. After the joining of expanded Lepidoptera, the level of dietary niche overlap among local Lepidoptera also dropped, indicating a decreased level of direct competition over food plants in both life stages. Thus, although the successfully expanded Lepidoptera surely brough about some previously inexistent resource competition to the communities, overall, they tended not to share as many food plants with the original residents as among the residents themselves. The negative impacts of Lepidoptera expansion appeared to be more pronounced on the plant assemblages than the Lepidoptera ones. Notably, these functional consequences were evaluated purely based on lepidoptera–plant interactions, but we cannot judge their relative importance when looking at a bigger picture where Lepidoptera’ and plants' interactions with many other taxa are also included; for instance, plants' competition for pollinating Lepidopterans may not be of high ecological importance if many other insects can help pollinate the same plants. Also, our simulative predictions emerged via assuming Lepidoptera have fixed diets throughout the space, yet we acknowledge that Lepidoptera's diet is partially plastic and can include novel plants (e.g., Graves & Shapiro, 2003). As such, our simulations may underestimate the impacts of Lepidoptera expansion. If the extent of utilising new food is somewhat predictable (e.g., Pearse & Altermatt, 2013b), it could be an extension of the current work to further incorporate the predicted food utilisation into the criteria for successful Lepidoptera expansion.

We used detailed lepidoptera–plant trophic reliance across both Lepidopteran life stages as a biotic filter predicting the viability of Lepidoptera to remain after local plant extinction or to establish in new sites after their expansion. This approach highlighted the fact that for holometabolous species, their different life stages may rely on non‐identical food resources, and corresponding food for each stage must be available to allow their completion of life cycle within a given space. We argue that such a consideration of the bio‐interaction context is needed whenever predicting species spatial distributional change (Robinson et al., 2011; Werner & Gilliam, 1984). We did not apply the same filter from the plants’ perspective, since to our knowledge, most of the flowering plants in our study area do not obligatorily rely on Lepidoptera's pollination to achieve their reproduction. Nonetheless, when addressing other animal–plant interaction systems where the reliance is more mutually important, e.g., between certain mistletoe and hummingbird species (Rodriguez‐Cabal et al., 2013), such a biotic filter may also matter to the plants’ viability at places.

Since our biological understanding lies in the study area, we could only detect the discontinuity of species distributions (at the resolution of our cell size) within this area, but whether such discontinuity would capture the Lepidopterans’ realistic “continental” thermal range boundaries was unknown. This may be particularly relevant as for the simulated northward expansion of Lepidoptera, as it is supposed to mimic the spatial shift of species’ cold boundaries in response to northern hemisphere's climate warming. We note that, however, if a Lepidopteran has its cold boundary further north from our study area, meanwhile its population widely distributed within, then its simulated northward expansion would basically trigger no compositional change to the cells. In other words, all consequences generated by our northward expansion simulations were driven by those Lepidopterans having northward discontinuity in their original “regional” distributions (i.e., having northward adjacent cell(s) without their occurrences). Such regional distributional discontinuity could be shaped by many factors, but most likely climatic and topographical ones, as a previous study in the same region (Ho et al., 2024) showed that local Lepidoptera richness is highly associated with temperature and elevation. Even for continentally widely distributed Lepidoptera species, it is possible that they form discontinuous (sub)populations, and each exhibit respective thermal range adapted to local climate (Bestion et al., 2015; Herrando‐Pérez et al., 2019). Thus, the northward expansion simulations may not be strictly realistic, yet is still relevant. On the other hand, the upward expansion simulations are more intuitively sensible, as literature have shown that the distributions of Lepidopterans are constrained by the elevational thermal gradient and responsive to climate change (Chen et al., 2009). Future studies could be expanded to include species’ continental ranges, possibly by utilising the occurrence data available from the Global Biodiversity Information Facility (GBIF; https://www.gbif.org). By doing so, the same questions as asked in the current study could be tackled but at a continental spatial scale, at which the species’ thermal ranges could be fully captured. Such data would be even less systematic, though, so one may need to compile the data to an even coarser level (e.g., spatially speaking, adopting a larger cell size) to control the uncertainties among different places. Alternatively, future studies could ask taxa‐oriented questions and thereby focusing on a subset of species for which more‐systematic and spatially better‐covered data are available (e.g., Neu et al., 2021).

In this study, we used Lepidoptera and flowering plants—predominant and well‐studied taxa in the terrestrial ecosystem—as the model system to predict potential biogeographic influences of global change. With the accumulation of bio‐interaction understanding, we have seen more and more metawebs becoming available recently (e.g., Gravel et al., 2019; O'Connor et al., 2020), covering other taxa and various ecosystems. A transition to study interaction networks in a spatially explicit (versus spatially implicit) approach is needed, as all interactions occur in a spatially structured world, exposed to global change‐associated shift in species' co‐occurrence. Our simulations integrate biogeographical and bio‐interaction knowledge, and provide prospects with mechanistic inferences of extinction‐ and expansion‐driven community change. This makes a step of advancement from previously spatially implicit explorations, and presents a paradigm shift of predictive biogeography that highlight the importance of integrating both the spatial and biological contexts of the focal processes (echoing Gravel et al., 2019; Thuiller et al., 2024; Tscharntke & Brandl, 2004). Given such an advancement, though, the current study had to adopt the spatial and temporal resolutions of the empirical data collected, and thus was operated at relatively coarse scales. Should finer‐scaled data become available, a more realistic approach mirroring the current study would be to fit species distribution models for all the focal species, then simulate their realistic distributional changes under detailed climate change scenarios (e.g., Adde et al., 2023); this approach would better benefit the scientific and applicative fields, arguably particularly the latter, as it could precisely inform the conservation of certain taxa at certain places. In this respect, the predictions of our simulative study help identify some penetrating points for empirical work. For instance, our simulations indicate that the Lepidoptera assemblages at the central sub‐region of Baden‐Württemberg are particularly prone to secondary extinctions. Empirical studies can thus prioritise the collection of high‐quality data in this sub‐region. In general, we call for future work to keep accumulating finely resolved spatio‐temporal data of species, or to revisit and recompile the currently available datasets to achieve better resolutions. We also encourage future work to keep generating the same predictive understanding of other biological systems by exploiting the distribution and interaction data available. We believe that the accumulation of such understanding will together lay an important foundation for the management of biodiversity under global change.

## AUTHOR CONTRIBUTIONS

**Hsi‐Cheng Ho:** Conceptualization (equal); data curation (equal); formal analysis (lead); funding acquisition (supporting); methodology (equal); visualization (lead); writing – original draft (lead); writing – review and editing (lead). **Florian Altermatt:** Conceptualization (equal); data curation (equal); funding acquisition (lead); methodology (equal); project administration (lead); supervision (lead); validation (lead); writing – original draft (supporting); writing – review and editing (supporting).

## FUNDING INFORMATION

Funding is through the University of Zurich Research Priority Program in Global Change and Biodiversity (URPP GCB) as well as the Swiss National Science Foundation (Grant Nr. 310030_197410) to FA. HH is supported by the Ministry of Education of Taiwan through the Yushan Young Fellow Program (Grant No. MOE‐112‐YSFAG‐0003‐005‐P1) and by the National Science and Technology Council of Taiwan (Grant No. NSTC 112‐2621‐B‐002‐006‐MY3).

## CONFLICT OF INTEREST STATEMENT

The authors claim that there are no conflicts of interest.

## Supporting information


Data S1.

## Data Availability

The Lepidoptera and plant occurrence data can be obtained at Database *Arbeitsgruppe Schmetterlinge Baden‐Württembergs*, *Bundesamt für Naturschutz* and *Floraweb*, respectively; the GIS data can be derived from *Shuttle Radar Topography Mission* (see Methods). The compiled metawebs can be accessed upon request to FA. The processed local community metrics (readings of each simulation), as well as the R script that reproduces all analyses and figures in this study, can be accessed at this public repository: https://doi.org/10.6084/m9.figshare.26625070.
