# Supplementary material for: Predicted community consequences of spatially explicit global change‐induced processes on plant–insect networks
Source: Ecol Evol. 2024 Sep 16;14(9):e70272. doi: 10.1002/ece3.70272 (PMC11405086; doi:10.1002/ece3.70272)

**Predicted community consequences of spatially explicit global change-induced processes on plant-insect networks**

**Supplementary Information**

**Section S1. Algorithm of isolation-driven plant extinction**

Here we verbally present the algorithm for performing isolation-driven plant extinction. The corresponding working R script can be found in the provided repository.

Starting from the compiled plant occurrence data, for each focal plant at each grid cell where it occurs, we calculated the cell-wise level of isolation as the ratio of “number of adjacent cells where this plant also occurs” to “number all adjacent cells”. Most commonly, this would be a value ranging between 0/8 (a fully isolated, single-cell population of the plant with no occurrence in any of the adjacent cells) and 8/8 (all eight adjacent cells have the same plant species occurring). For cells that are located on the boundaries of the study area, because we did not know plant occurrence outside the study area, we accordingly only accounted for adjacent cells that are also within the study area. For example, an edge cell having only five adjacent cells with known plant information will have a level of isolation derived between 0/5 and 5/5. Based on this evaluated level of isolation of each cell where the focal plant occurs, we then removed the plant’s occurrence at the most isolated cells (i.e., a derived value closest to zero; those having the values were removed altogether). The level of isolation of all remaining cells where the focal plant remained occurring was re-evaluated again (note that compared to before removal, the level of some cells will change). This process is repeated until the plant is fully removed from the study area, i.e., occupying no cell at all. In effect, this procedure makes isolated and periphery local populations (defined at the cell-size scale) of a plant extinct earlier than the central ones. Also, for each focal plant, we derived a spatially explicit extinction sequence encoding how each step of cell-wise removal makes the plant gradually disappear in the study area.

The number of removals (cell-wise extinctions) required for making a plant fully extinct in the study area differs from one plant species to another, according to their original ways of distributing. A very broadly distributed plant may take more than 200 cell-wise removal to become fully removed. To make our simulation efficient and practical, we coarsened the total number of removals required per plant by rounding it up and making it in proportion to the square root of the plant’s original number of cells occupied. Thus, if a plant occupies a four times larger spatial area (in terms of cells) than another plant, the former will require (roughly) two times more removal steps than the latter. The most broadly distributed plant in our data basically covered the whole area (302 cells), and by taking the square root of such cell occupancy, such a plant required in total of eighteen extinction steps to become regionally extinct (at each step multiple cell-wise extinctions can happen); all others required equal or fewer, and some scattered and very isolated plants get fully removed in only one step.

Up to this step, we derived for all focal plants how many isolation-driven extinction steps they needed to get regionally extinct, and for each extinction step how their cell-wise occupancy will change. For the twenty independent simulations of isolation-driven random scheme, each time we just randomly rearranged the sequence of all these extinction steps, so that the local extinction of every plant interspersed each other, and we evaluated the response metrics after each step. For the isolation-driven status-weighted scheme, all else was equal, yet the sequence of all extinction steps was not fully randomly arranged, but weighted by the plants’ status (see below, section S2) so that highly threatened plants tended to have extinction steps happen earlier in the simulation.

**Section S2. Additional information of the plant data**

Besides the spatial occurrence data of plants from the respective databases as specified in main-text Methods, we also compiled the plants’ status of being threatened within the study area. The status data of the focal plants were derived mainly from Breunig, T. & Demuth S. (1999): Rote Liste der Farn- und Samenpflanzen Baden-Württemberg, by Landesanstalt für Umweltschutz, Germany. Based on this data source, we defined the status of the focal plants in Baden-Württemberg into one of the following categories:

| Category | Status/description |
| --- | --- |
| 0 | Extinct or disappeared |
| 1 | Threatened with extinction |
| 2 | Highly endangered |
| 3 | Endangered |
| G | Endangered yet risk level unclear |
| R | Extremely rare |
| V | Early warning |
| d | Data insufficient |
| * | Not endangered |

Such compiled status data covered 714 out of the 848 focal plants (after synonyms were unified). We then complemented the rest based on information from online databases available, including:

- <https://www.infoflora.ch/>
- <https://www.iucnredlist.org/>
- [https://wfoplantlist.org/](https://wfoplantlist.org/plant-list/taxon/)
- <http://www.theplantlist.org/>

From the information in these online databases, plants were assigned a status following IUCN red list coding. Some information was from a global perspective, not necessarily at the scale of Baden-Württemberg, yet we adopted it. By the end of this procedure, all focal plants were assigned a respective status of being threatened. Then, we roughly grouped the status of all focal plants into “threatened” or “not threatened”: the latter included the “*” and “d” categories defined in Breunig & Demuth (1999), as well as “Least-Concern”, “Near-Threatened”, “Unknown (incl. data deficient and not evaluated ones)” categories from the online databases; the former included all the rest, integrating all levels of being threatened. For ease of our simulation purpose, we settled with these two rough categories of “threatened” vs. “not threatened” but not more detailed levels.

For the “status-weighted” plant extinction schemes, the full extinction sequence—i.e., at each step which plant species being picked to get removed (at certain cells if it was isolated-driven schemes, too)—is composed stochastically from the first to the last extinction event, yet the threatened species were weighted five times more than the not-threatened ones. Thus, in effect, the extinction of threatened plants tended to happen earlier than those of the not-threatened plants.

**Section S3. Additional information of the Lepidoptera data**

Besides the spatial occurrence data of our focal Lepidoptera, we also extracted information of their morphology also from Ebert, G. (1991–2005). Die Schmetterlinge Baden-Württembergs. Vols. 1–10. These data were measured with Lepidoptera individuals collected within the study area. The species-specific measurements were based on one individual only, thus we acknowledged the morphological information was somewhat undersampled; nonetheless we embraced the advantage that the data were empirically derive and had excellent taxonomic coverage.

To derive the flying ability of each Lepidoptera species, we first derived their wing load from the morphological data. In the data, the width and length (in cm) of individual’s body and wing were available. We thus first approached individual’s body volume as the square of body width multiplied by body length. Then, by assuming such body volume is an unbiased estimate of body weight (in arbitrary unit), we divided the body volume by the wing area, which was the product of wing length and width, to get an estimated wing load for each focal Lepidoptera. The wing load readings were then normalised to the minimal reading among all.

We assumed that the insects’ flying ability is inversely associated with their wing loads (i.e., the smaller the wing load, the better the flying ability), and better fliers have higher expanding potentials. Thus, in our simulations of Lepidoptera expansion, in “wingload-weighted” schemes, the Lepidoptera to be expanded (drawn at each simulative step) were picked not purely randomly, but based on probabilities weighted inversely with the Lepidoptera’s normalised wing load readings (yet the picking was still a stochastic process). Species with low wing load, thus supposedly better flying ability, had a higher probability to picked and set expanding.

**Section S4. Additional figures & tables**


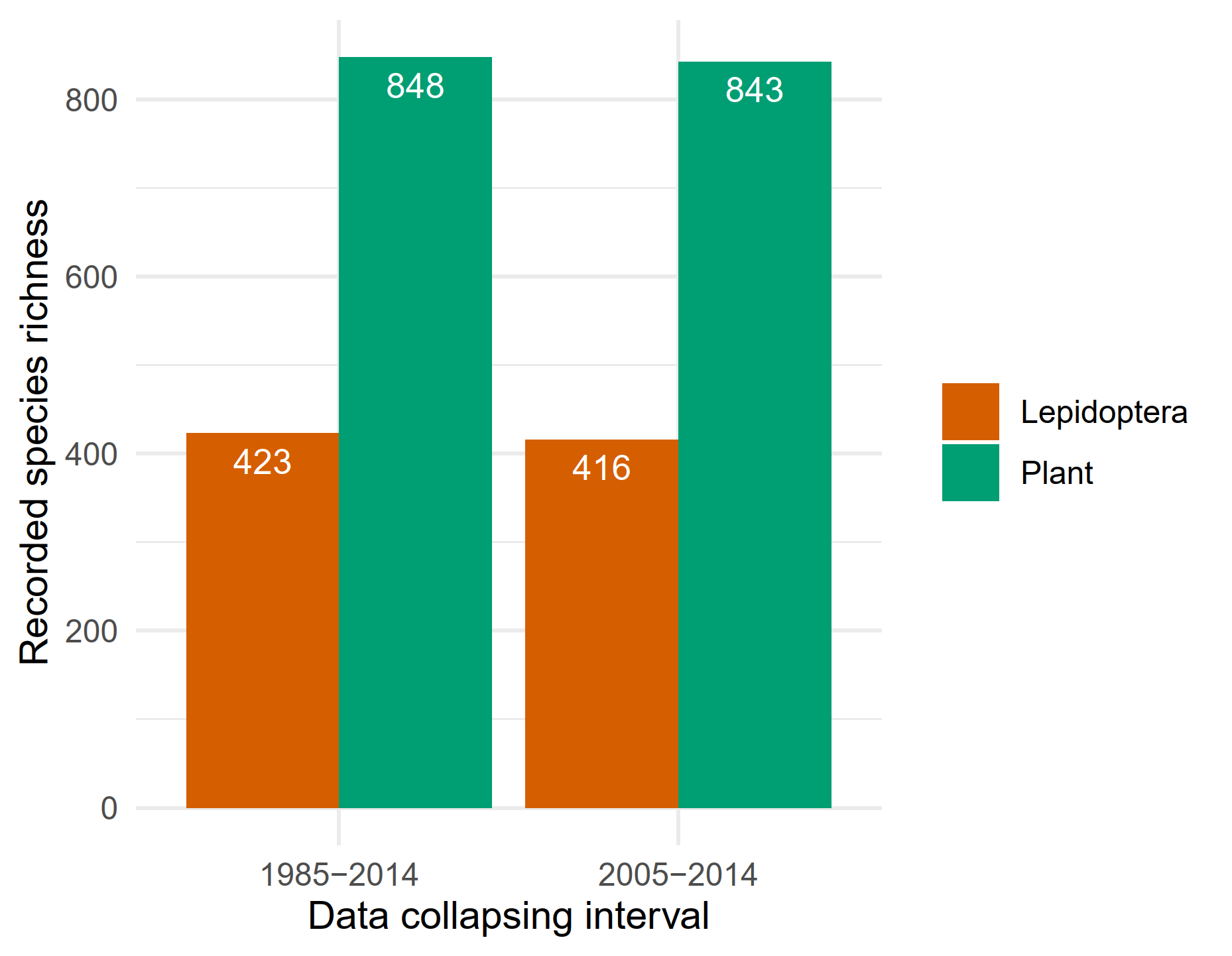


Figure S1. Recorded Lepidoptera and plant species richness within the study area based on empirical occurrences, compared between two data-collapsing intervals. The 1985-2014 is our applied default interval, whereas the 2005-2014 represents a narrower, one-decade interval that is closer to the near recent.


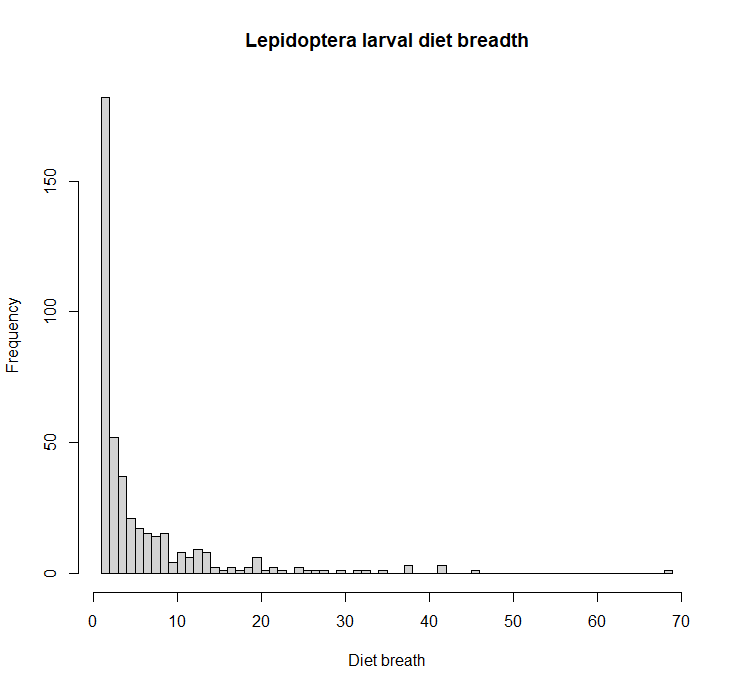

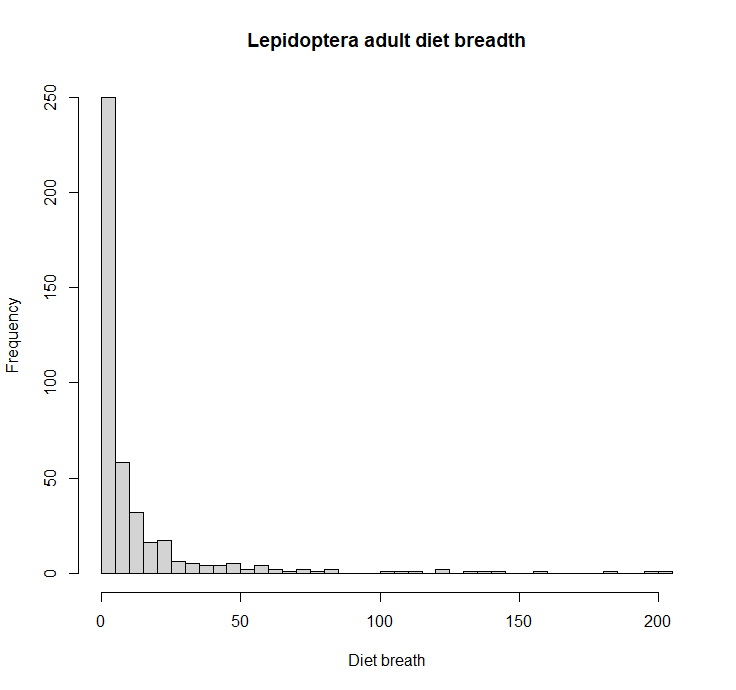


Figure S2. The potential diet breadths of larval and adult Lepidoptera considered in this study. The potential diet breaths refer to number of edible food plants listed in the metaweb of each Lepidopteran.

**
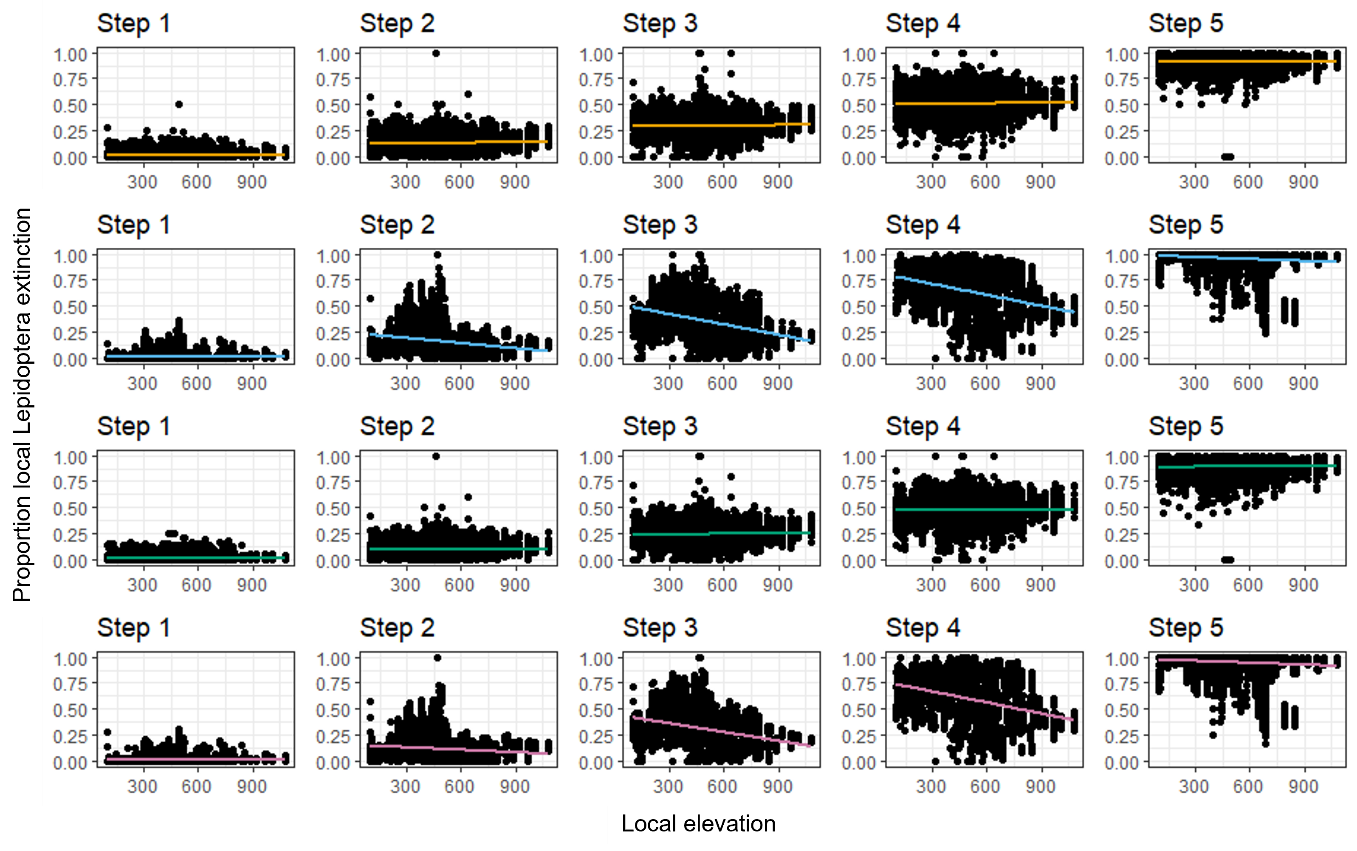
**

Figure S3. Proportion local Lepidoptera secondary extinction against the cell’s local mean elevation in the four schemes of plant extinction simulation, at five selected time points as columns (time points corresponding to Fig. 1). Simulation schemes are as rows, from top to bottom: Regional Random, Isolation-driven Random, Regional Status-weighted, and Isolation-driven Status-weighted. The trends were visualised using linear regression (coloured lines and shaded 95% CIs, across 20 replicates), while corresponding stats (including alternatives with mixed model setting replicates as a random effect) are provided in Table S1. Main-text Fig. 3 was plotted based on the 4^th^ column here.

**
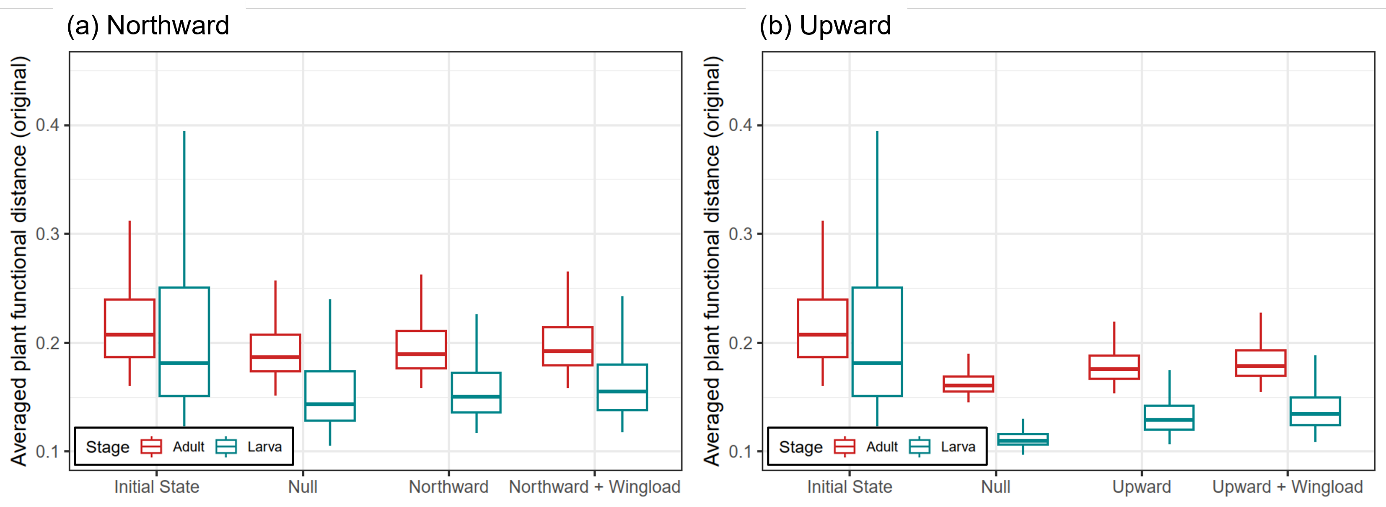
**

Figure S4. Averaged plant functional distance in the local interaction networks formed with adult and larval Lepidoptera (indicated with colours) at the initial state (before simulation) and after three steps of Lepidoptera expansion simulation. (a) panel shows northward, northward wingload-weighted, and corresponding null schemes, and (b) panel shows the upward ones. The boxplots indicate the median (middle thick line), the 1st and 3rd quantiles (Q1 and Q3, the hinges of the box), and the smallest/largest value (the whiskers) within Q1-1.5×IQR and Q3+1.5×IQR (where IQR = Q3-Q1), respectively.


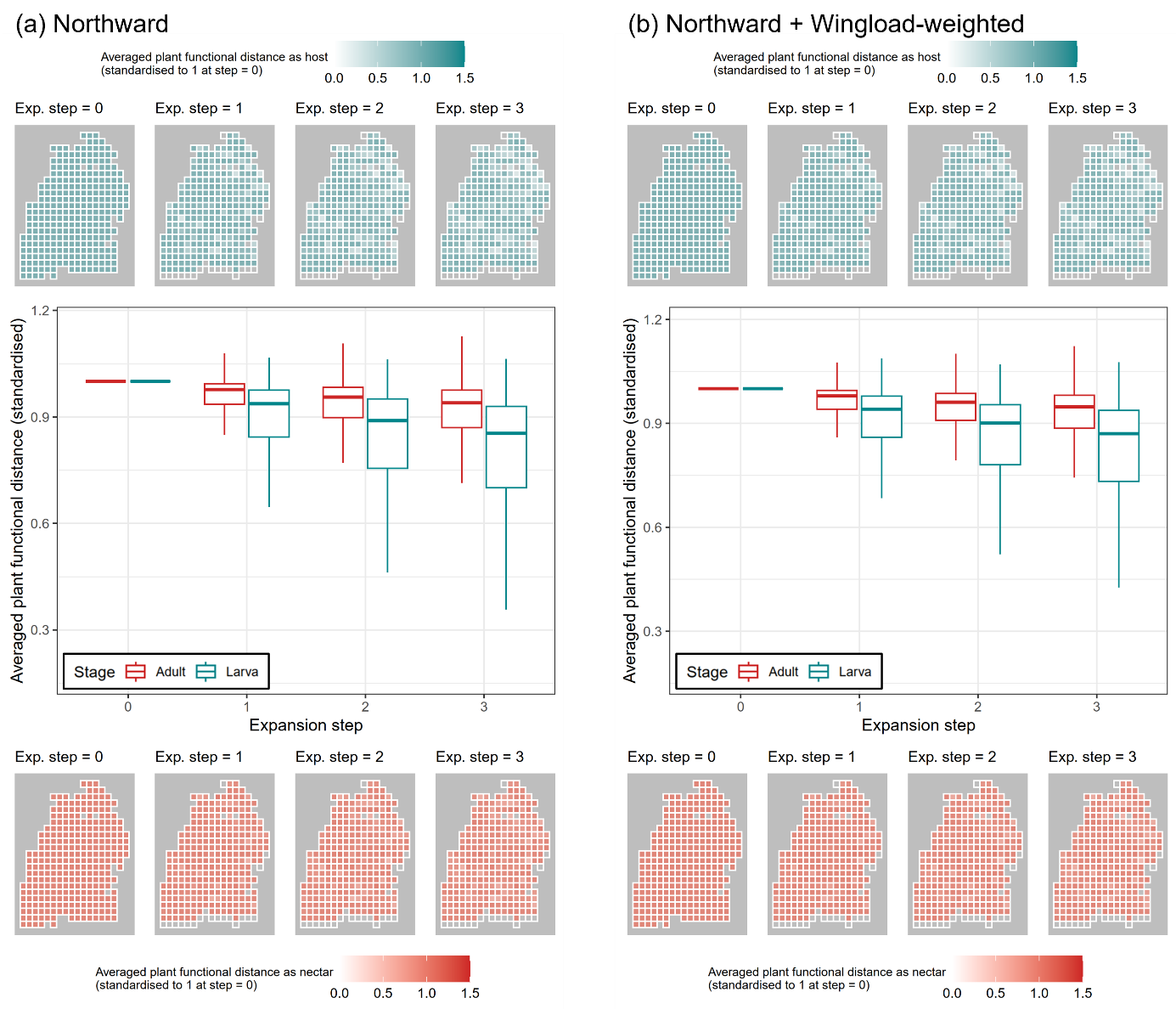


Figure S5. Averaged plant functional distance in the local interaction networks formed with adult and larval Lepidoptera (indicated with colours) at the initial state (before simulation) and the three steps of Lepidoptera expansion simulation of (a) northward and (b) Northward Wingload-weighted schemes. The readings were standardised by dividing by the value at the initial state to highlight the relative changes. The boxplots indicate the median (middle thick line), the 1st and 3rd quantiles (Q1 and Q3, the hinges of the box), and the smallest/largest value (the whiskers) within Q1-1.5×IQR and Q3+1.5×IQR (where IQR = Q3-Q1), respectively. The maps above and below the boxplots show the how the cell-wise readings of larval and adult networks change throughout the simulation, respectively. Note that we excluded cells with too small plant or Lepidoptera species number (<10) from the analyses since functional distance evaluation in small networks can be highly biased. Also, after simulation started, we did not account for the cells where the Lepidoptera composition is not influenced by Lepidoptera expansion, i.e., those without further-south data-available cells. These cells were coloured grey in the maps.


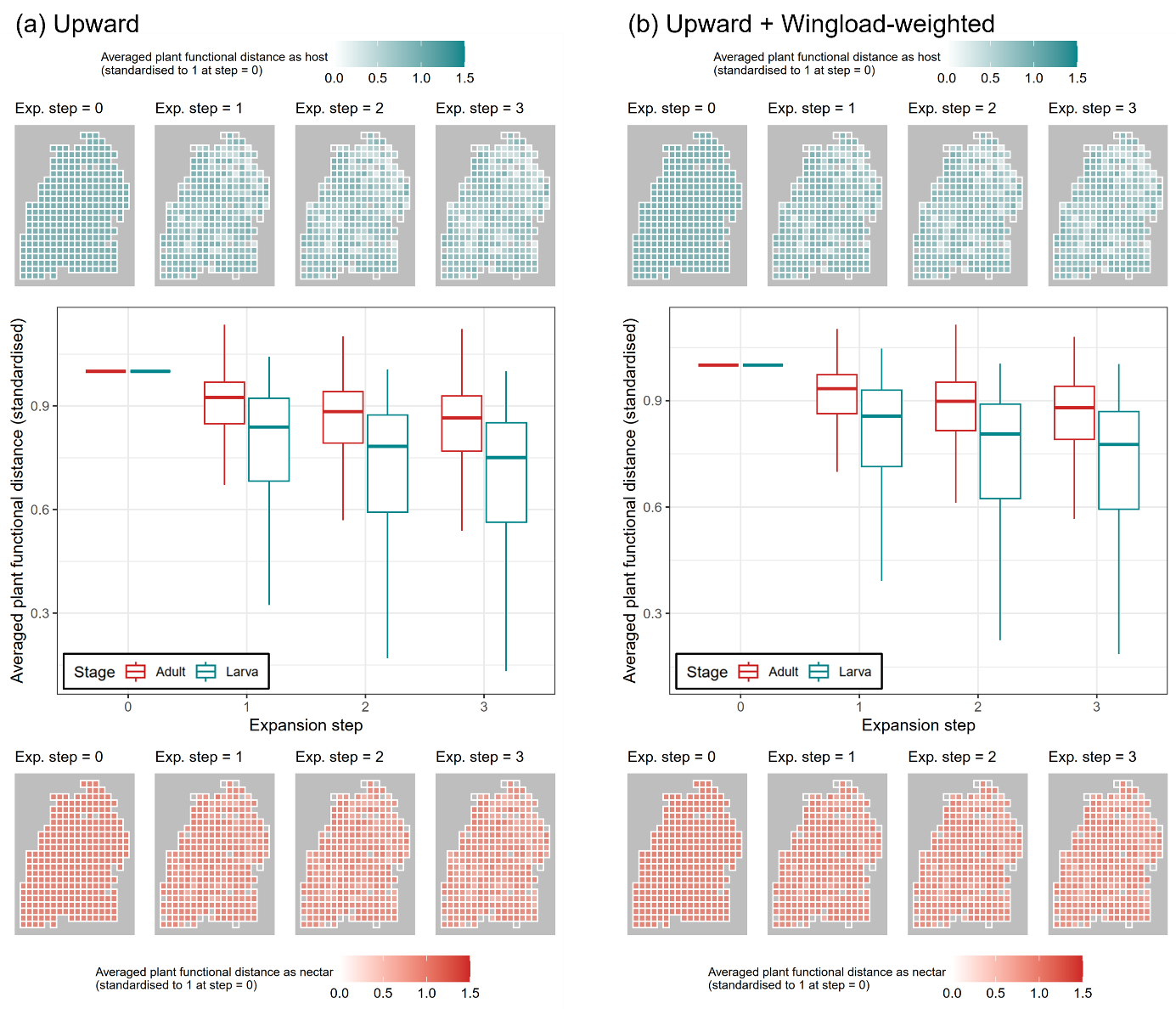


Figure S6. Averaged plant functional distance in the local interaction networks formed with adult and larval Lepidoptera (indicated with colours) at the initial state (before simulation) and the three steps of Lepidoptera expansion simulation of (a) upward and (b) Upward Wingload-weighted schemes. The readings were standardised by dividing by the value at the initial state to highlight the relative changes. The boxplots indicate the median (middle thick line), the 1st and 3rd quantiles (Q1 and Q3, the hinges of the box), and the smallest/largest value (the whiskers) within Q1-1.5×IQR and Q3+1.5×IQR (where IQR = Q3-Q1), respectively. The maps above and below the boxplots show the how the cell-wise readings of larval and adult networks change throughout the simulation, respectively. Note that we excluded cells with too small plant or Lepidoptera species number (<10) from the analyses since functional distance evaluation in small networks can be highly biased. Also, after simulation started, we did not account for the cells where the Lepidoptera composition is not influenced by Lepidoptera expansion, i.e., those without lower adjacent data-available cells. These cells were coloured grey in the maps.

**
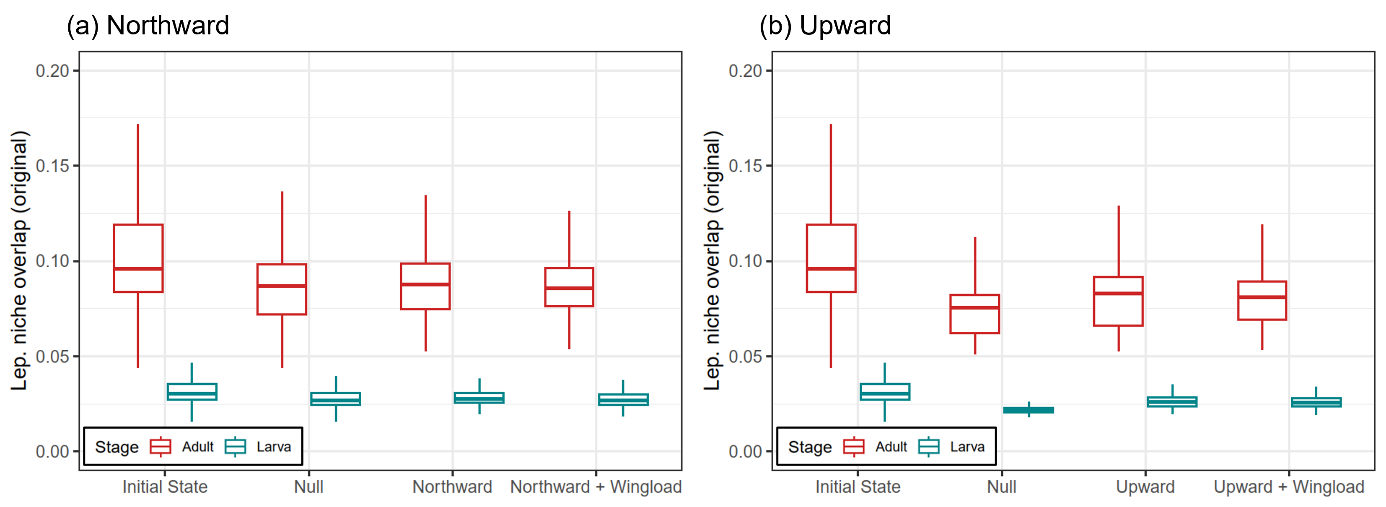
**

Figure S7. Averaged adult and larval (indicated with colours) Lepidoptera dietary niche overlap in the local interaction networks formed with food plants at the initial state (before simulation) and after three steps of Lepidoptera expansion simulation. (a) panel shows northward, northward wingload-weighted, and corresponding null schemes, and (b) panel shows the upward ones. The boxplots indicate the median (middle thick line), the 1st and 3rd quantiles (Q1 and Q3, the hinges of the box), and the smallest/largest value (the whiskers) within Q1-1.5×IQR and Q3+1.5×IQR (where IQR = Q3-Q1), respectively.


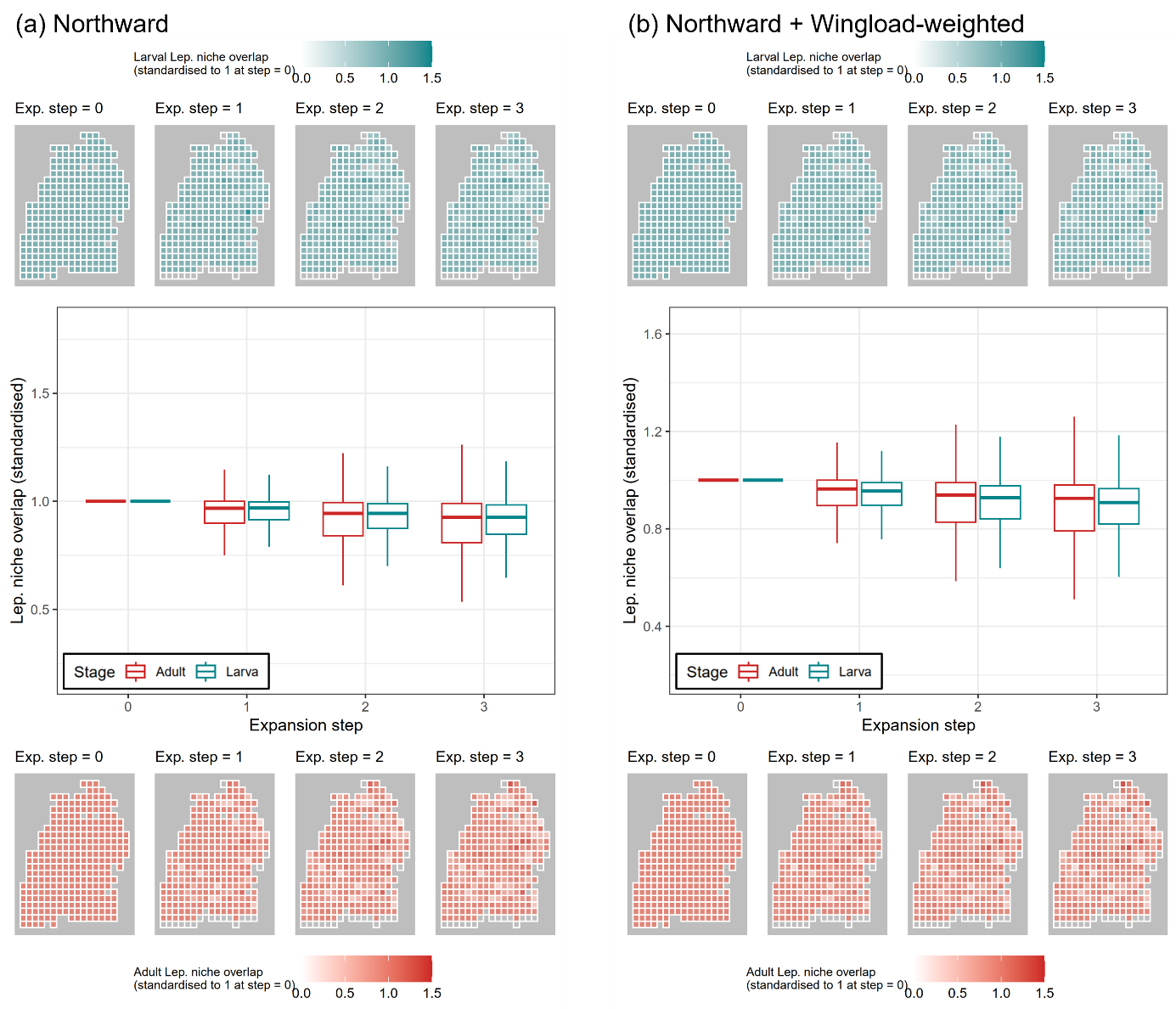


Figure S8. Averaged adult and larval (indicated with colours) Lepidoptera dietary niche overlap in the local interaction networks formed with food plants at the initial state (before simulation) and the three steps of Lepidoptera expansion simulation of (a) Northward and (b) Northward Wingload-weighted schemes. The readings were standardised by dividing by the value at the initial state to highlight the relative changes. The boxplots indicate the median (middle thick line), the 1^st^ and 3^rd^ quantiles (Q1 and Q3, the hinges of the box), and the smallest/largest value (the whiskers) within Q1-1.5×IQR and Q3+1.5×IQR (where IQR = Q3-Q1), respectively. The maps above and below the boxplots show the how the cell-wise readings of larval and adult networks change throughout the simulation, respectively. Note that we excluded cells with too small plant or Lepidoptera species number (<10) from the analyses since functional distance evaluation in small networks can be highly biased. Also, after simulation started, we did not account for the cells where the Lepidoptera composition is not influenced by Lepidoptera expansion, i.e., those without further-south data-available cells. These cells were coloured grey in the maps.


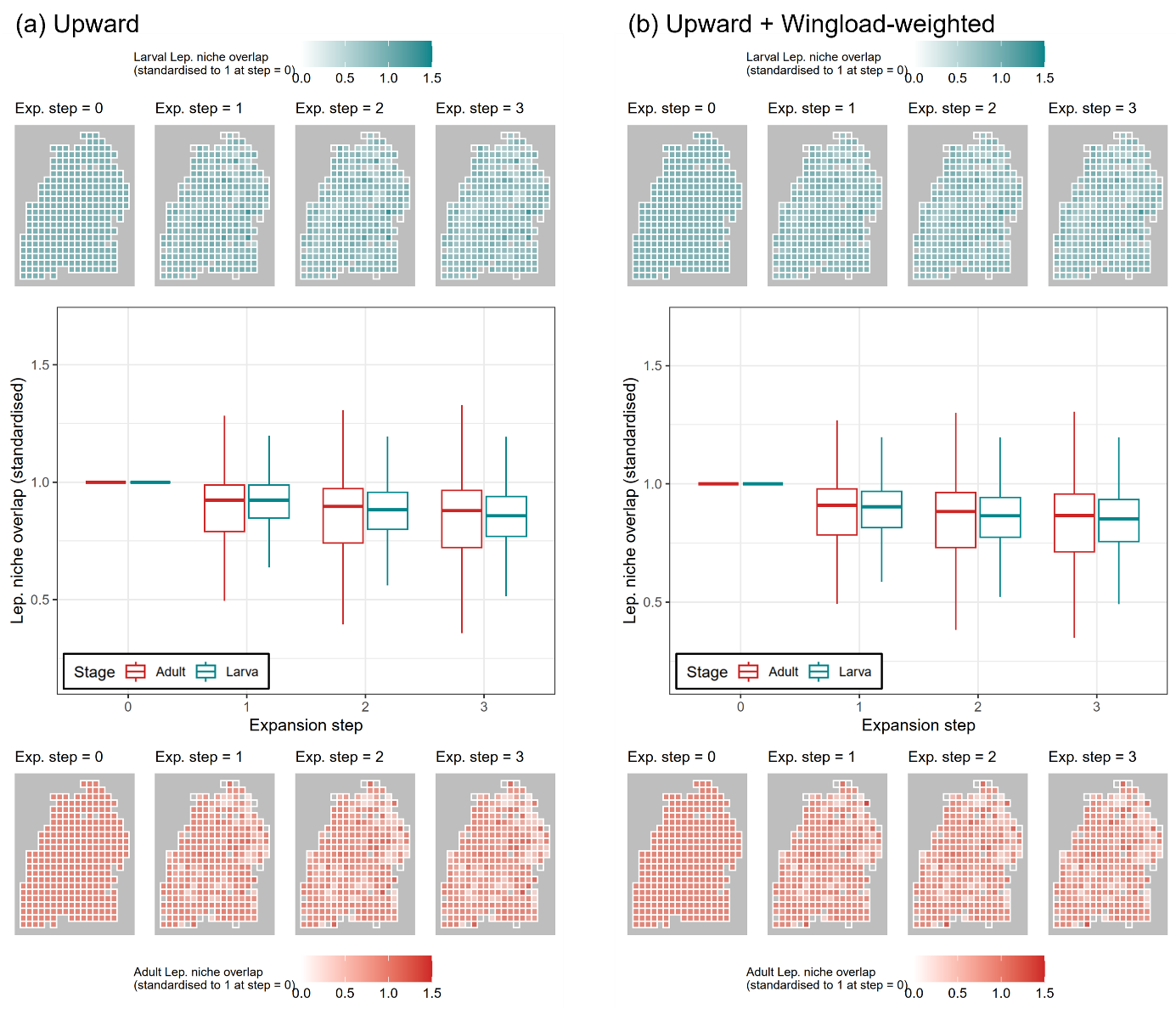


Figure S9. Averaged adult and larval (indicated with colours) Lepidoptera dietary niche overlap in the local interaction networks formed with food plants at the initial state (before simulation) and the three steps of Lepidoptera expansion simulation of (a) Upward and (b) Upward Wingload-weighted schemes. The readings were standardised by dividing by the value at the initial state to highlight the relative changes. The boxplots indicate the median (middle thick line), the 1^st^ and 3^rd^ quantiles (Q1 and Q3, the hinges of the box), and the smallest/largest value (the whiskers) within Q1-1.5×IQR and Q3+1.5×IQR (where IQR = Q3-Q1), respectively. The maps above and below the boxplots show the how the cell-wise readings of larval and adult networks change throughout the simulation, respectively. Note that we excluded cells with too small plant or Lepidoptera species number (<10) from the analyses since functional distance evaluation in small networks can be highly biased. Also, after simulation started, we did not account for the cells where the Lepidoptera composition is not influenced by Lepidoptera expansion, i.e., those without lower adjacent data-available cells. These cells were coloured grey in the maps.


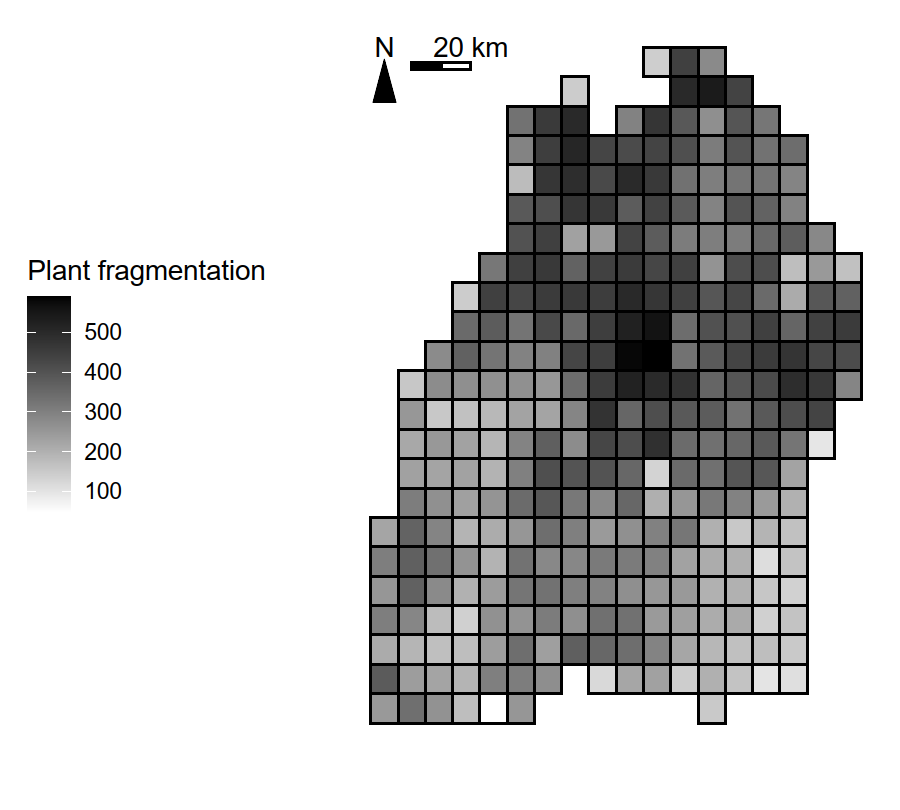


Figure S10. The level of distributional fragmentation of the focal plants in the study area at the initial state (before simulation) given by empirical data. This was judged according to the cell-wise extinction events in the isolation-driven plant extinction scheme, where, for each plant, each of its occupied cells was assigned a number in the extinction sequence (1 indicates the where the first local extinction will happen, at the most isolated/peripheral cell, and 2 the next one, so on and so forth). We here accounted for the cells per plant where the number was smaller than or equal to 70 (roughly 25% of the total number of cells, thus representing relatively isolated/peripheral cells), then summed the counting across all the 848 plants to derive the plant fragmentation index as indicated in this figure—a higher index value indicates more plant species being isolated/peripheral in a given cell. We note central Baden-Württemberg generally possessed higher values.

.

Table S1. Stats of the regressions performed for analysing proportion local Lepidoptera secondary extinction against the cell’s local mean elevation in the four schemes of plant extinction simulation, at five selected time points (corresponding to Fig. S3). Two regression models were included: simple linear regression (lm), and a mix model (lmm) where the 20 replicates of each simulation scheme were set as the random effect. We note that, in isolation-driven schemes, a negative slope soon became detectable (and is several orders more pronounced than any slopes in the regional schemes) after the simulation started, indicating Lepidoptera’s secondary extinction happened earlier at low-elevation grid cells.


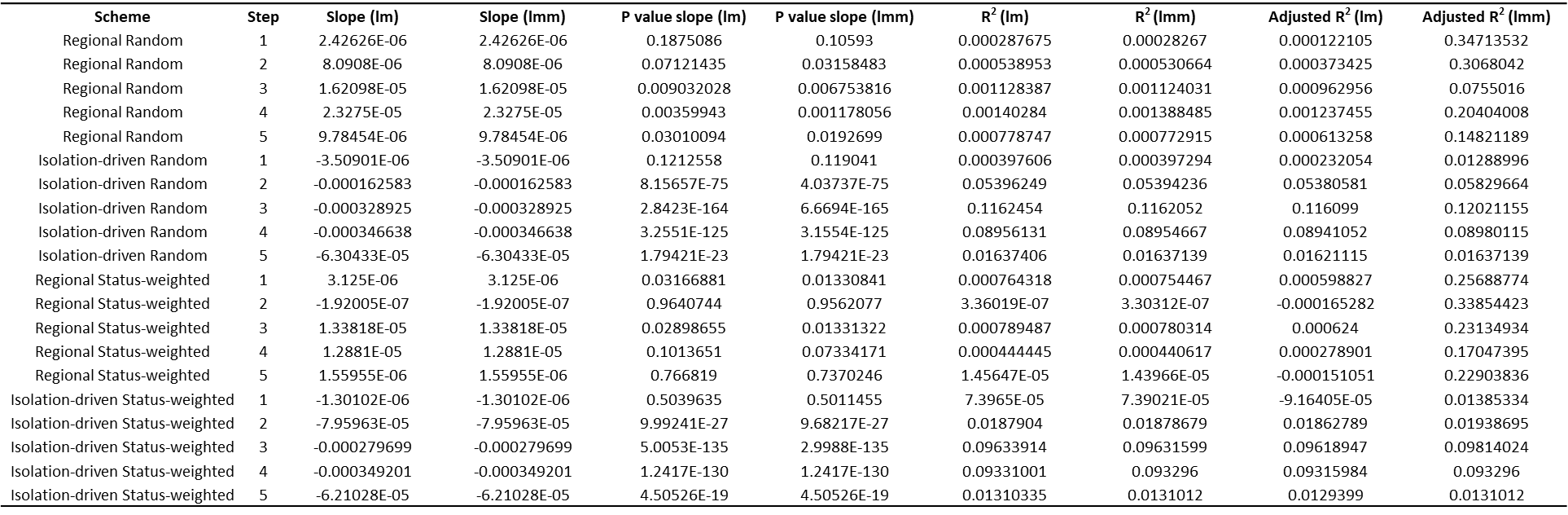

Supplement: Supplementary file 1 — Data S1. [file ECE3-14-e70272-s001.docx]
